# Identification of the common differentially expressed genes and pathogenesis between neuropathic pain and aging

**DOI:** 10.3389/fnins.2022.994575

**Published:** 2022-10-19

**Authors:** Qingqing Ye, Zhensheng Huang, Weicheng Lu, Fang Yan, Weian Zeng, Jingdun Xie, Weiqiang Zhong

**Affiliations:** State Key Laboratory of Oncology in Southern China, Department of Anesthesiology, Collaborative Innovation for Cancer Medicine, Sun Yat-sen University Cancer Center, Guangzhou, China

**Keywords:** neuropathic pain, aging, co-DEGs, immune infiltration, regulation network

## Abstract

**Background:**

Neuropathic pain is a debilitating disease caused by damage or diseases of the somatosensory nervous system. Previous research has indicated potential associations between neuropathic pain and aging. However, the mechanisms by which they are interconnected remain unclear. In this study, we aim to identify the common differentially expressed genes (co-DEGs) between neuropathic pain and aging through integrated bioinformatics methods and further explore the underlying molecular mechanisms.

**Methods:**

The microarray datasets GSE24982, GSE63442, and GSE63651 were downloaded from Gene Expression Omnibus (GEO) database. Differentially expressed genes (DEGs) and co-DEGs were first identified. Functional enrichment analyses, protein-protein Interaction (PPI) network, module construction and hub genes identification were performed. Immune infiltration analysis was conducted. Targeted transcription factors (TFs), microRNAs (miRNAs) and potential effective drug compounds for hub genes were also predicted.

**Results:**

A total of 563 and 1,250 DEGs of neuropathic pain and aging were screened, respectively. 16 genes were further identified as co-DEGs. The functional analysis emphasizes the vital roles of the humoral immune response and complement and coagulation cascades in these two diseases. Cxcl14, Fblim1, RT1-Da, Serping1, Cfd, and Fcgr2b were identified as hub genes. Activated B cell, mast cell, activated dendritic cell, CD56 bright natural killer cell, effector memory CD8 + T cell, and type 2 T helper cell were significantly up-regulated in the pain and aging condition. Importantly, hub genes were found to correlate with the activated B cell, activated dendritic cell, Gamma delta T cell, central memory CD4 + T cell and mast cell in pain and aging diseases. Finally, Spic, miR-883-5p, and miR-363-5p et al. were predicted as the potential vital regulators for hub genes. Aldesleukin, Valziflocept, MGD-010, Cinryze, and Rhucin were the potential effective drugs in neuropathic pain and aging.

**Conclusion:**

This study identified co-DEGs, revealed molecular mechanisms, demonstrated the immune microenvironment, and predicted the possible TFs, miRNAs regulation networks and new drug targets for neuropathic pain and aging, providing novel insights into further research.

## Introduction

Neuropathic pain is a debilitating neurobiological disease caused by damage or diseases of the somatosensory nervous system and characterized by spontaneous pain or amplified pain responses (Jensen et al., 2011). It is a major socioeconomic problem that affects 7–10% of the population (van Hecke et al., 2014). Patients with neuropathic pain are often accompanied by sleep disturbances, depression, and severe impairment of daily life (Finnerup et al., 2021). Its prevalence has been projected to increase with the aging population, which is directly associated with suffering, disability, and higher costs to health care systems (Domenichiello and Ramsden, 2019).

Aging is a progressive loss of physiological integrity that leads to diminished function and increased mortality (López-Otín et al., 2013). It is one of the leading risk factors for most neurodegenerative diseases, including Alzheimer’s disease (AD) and Parkinson’s disease (PD) (Hou et al., 2019). Numerous studies have also indicated the strong relationship between neuropathic pain and aging. Persistent pain has been associated with aging (Saraiva et al., 2018), and aging may contribute to descending inhibition of pain pathways (Karp et al., 2008). Additionally, older adults with pain conditions are more likely to age than pain-free individuals (Karp et al., 2008). However, the underlying mechanisms involved in neuropathic pain and aging remain elusive.

In recent years, advances in microarray chip technology and other bioinformatics tools have made it possible to explore the common molecular mechanisms of disease–disease interaction. Here, We explored common differentially expressed genes (co-DEGs) and underlying molecular mechanisms between neuropathic pain and aging models. Three gene expression datasets (GSE24982, GSE63442, and GSE63651) were downloaded from the Gene Expression Omnibus (GEO) database. Through integrated bioinformatics analyses, co-DEGs and their functional roles in neuropathic pain and aging were identified. PPI network, critical modules and hub genes were predicted. The immune microenvironment was further revealed, and the potential roles of hub genes in immune regulation were demonstrated. Finally, we investigated these genes’ TFs, associated miRNAs and predicted possible effective target drug compounds. The present study sought to provide new insights into the molecular mechanisms underlying neuropathic pain and aging.

## Materials and methods

### Data acquisition

Three microarray datasets were first downloaded and analyzed from the GEO database.^1^ The GSE24982 dataset contains 10 pairs of contralateral and 10 pairs of ipsilateral L4 or L5 Dorsal Root Ganglion (DRG) tissue samples with sham surgery or Spinal Nerve Ligation (SNL) surgery (von Schack et al., 2011). GSE63442 dataset consists of 6 sham surgery samples and 6 SNL surgery samples from the rat DRG tissue. GSE63651 consists of 4 young (6 months-old) normal-fed rats,4 old (25–28 months-old) normal-fed rats, and 4 old (25–28 months-old) fed restrictedly. We further selected 10 ipsilateral post-sham DRG tissue in the GSE24982 dataset as the control group and 10 ipsilateral SNL post-op DRG tissue as the experimental group. The six sham-operated DRG samples in the GSE63442 dataset were regarded as the control group, and the other six SNL postoperative DRG samples were regarded as the experimental group. The four young samples of GSE63651 were used as the control group, and the four old samples were used as the experimental group. All three datasets were generated from transcriptome microarray arrays.

### Identification of differentially expressed genes and common differentially expressed genes

Using the R language, we normalized the raw data using the normalizeBetweenArrays function, in which the data would be log2 transformed if needed. Differentially expressed genes (DEGs) were identified based on R packages “limma” and “impute” (Ritchie et al., 2015; Hastie et al., 2021). DEGs were defined as genes with adjusted *P*-value < 0.05 and | logFC (fold change) | ≥ 0.58 in the pain datasets, and genes with *p*-value < 0.05 and | logFC (fold change) | ≥ 0.58 in the aging dataset. After screening the DEGs, we first intersected the two pain datasets to collect intersecting genes and then identify the overlapping genes among neuropathic pain and the aging dataset. Genes with opposite expression trends between neuropathic pain and aging datasets were removed.

### Tissue-most expressed gene analysis

We next identified the most related systems/tissues in neuropathic pain and aging by exploring co-DEGs’ distribution. We used the online resource BioGPS^2^ to analyze the most related systems/tissues with high expression of the co-DEGs. The most related systems/tissues should be the top two expressed systems/tissues, which can be identified as having a certain degree of specificity: (1) the most related tissues-expression level was more than the three multiples of the median, and (2) the second related tissue expression was more than the two multiples of the median.

### Functional enrichment pathway analysis

We performed Gene Ontology (GO) and Kyoto Encyclopedia of Genes and Genomes (KEGG) pathway analyses by “clusterProfiler” R package on the intersection DEGs of GSE24982 and GSE63442, the DEGs of GSE63651, and the co-DEGs of the two phenotypes (Wu et al., 2021). Results with *P*-value < 0.05 were indicated as significant. The “ggplot2” package was used to draw the bubble chart (Wickham, 2016). Gene Set Enrichment Analysis (GSEA) was further performed to validate the significant pathway based on GSE24982, GSE63442, and GSE63651, respectively (Subramanian et al., 2005).

### Construction of protein-protein interaction network

We applied the GeneMANIA online database^3^ to predict co-DEGs’ PPI network (Warde-Farley et al., 2010). It is an online analysis tool that widely used to demonstrate and evaluate gene interactions (Jiang et al., 2022; Tian et al., 2022). Given a query list of genes, GeneMANIA will find more genes like those. Most importantly, with a long enough list (currently five or more genes), GeneMANIA will weight data sources based on their predictive value for reconstructing the query list by default (Franz et al., 2018). We searched the 16 co-DEGs in GeneMANIA with the search parameters of the organism as Rattus-norvegicus and network weighting as an automatically selected weighting method. The network was then imported into Cytoscape (Version: 3.7.1) (Shannon et al., 2003) for visualization. To identify the interaction between co-DEGs, we further extracted co-DEGs networks with interactions from the PPI network. The cytoHubba plug-in was then applied to identify the hub genes based on co-DEGs in Cytoscape using the degree algorithm. In addition, the Minimal Common Oncology Data Elements (MCODE) plug-in was used to identify the potential vital modules. Default criteria in MCODE plug-in were set as follows: Degree Cutoff = 2, Node Score Cutoff = 0.2, K-Core = 2, and Max. Depth = 100.

### Immune infiltration analysis

ssGSEA (single-sample GSEA) algorithm was applied to evaluate the immune infiltration in pain and aging based on GSE24982 and GSE63651. It is a rank-based method that defines a score representing the degree of absolute enrichment of a particular gene set in each sample (Bindea et al., 2013; Finotello and Trajanoski, 2018). The correlations between co-DEGs and 28 immune cells were further determined using Spearman correlation analysis (Zhang et al., 2020a).

### Identification of transcription factors and microRNAs

TRRUST^4^ is a database for predicting transcription factors. We obtained hub genes’ TFs through this database. After downloading the results, we constructed a TF-gene interaction network in Cytoscape. The website miRDB^5^ is a comprehensive database of microRNAs. Compared with other databases, the microRNAs collected by miRDB are more complete and fully annotated. Through the Target mining module, we submitted hub genes as gene targets and then downloaded predicted miRNAs with scores greater than 50 points. The results were also visualized by Cytoscape.

### Drug prediction of hub genes

The drug prediction database DGIdb^6^ can predict gene-related drugs. We used the drug-gene interaction search module with parameters (source database = 22, gene category = 43, interaction type = 31) to predict drugs for the hub genes. The Cytoscape software conducted further visualization.

## Results

### Identification of differentially expressed genes in neuropathic pain and aging

**FIGURE 1:**
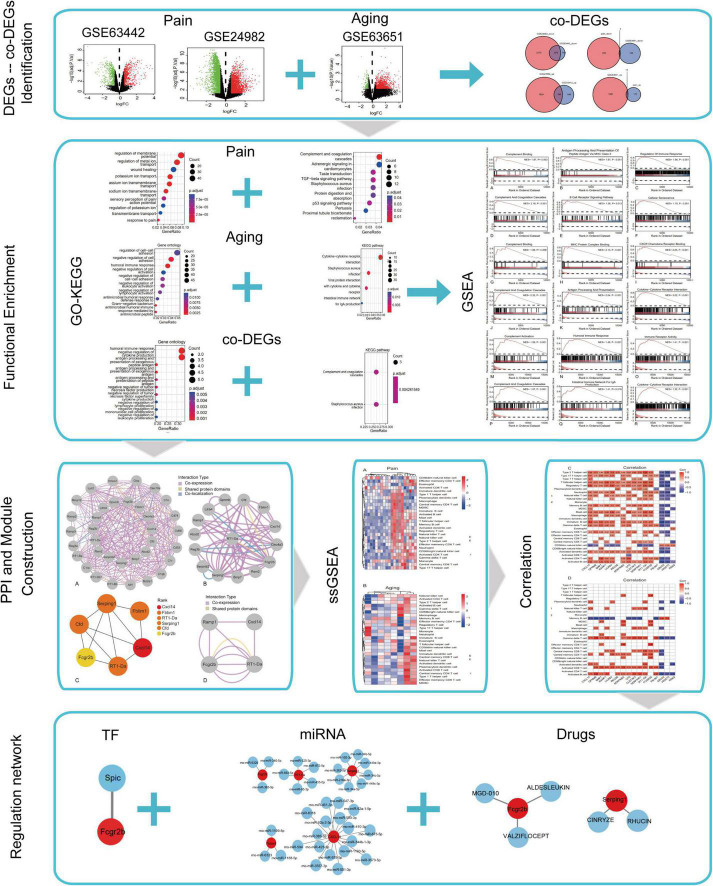
Flow chart of this study.

**FIGURE 2:**
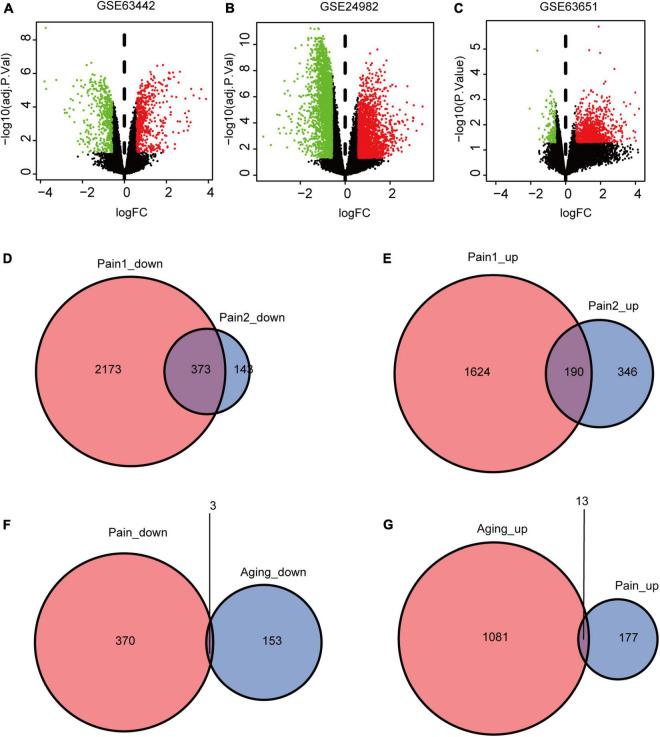
DEGs and co-DEGs identification. **(A–C)** The volcano plots of pain1: GSE63442, pain2: GSE24982 and aging: GSE63651. **(D,E)** The two pain datasets showed an overlap of 373 down-regulated DEGs and 190 upregulated DEGs. **(F,G)** The two neuropathic pain and aging datasets showed an overlap of 16 co-DEGs (3 down-regulated and 13 upregulated). DEGs, differential expressed genes; co-DEGs, common differential expressed genes.

The flowchart of this research is shown in Figure 1. We first downloaded two pain datasets numbered GSE24982, GSE63442, and an aging dataset GSE63651 from the GEO database. According to the previously set criteria, we first identified the DEGs of the two pain datasets (Figures 2A,B). A total of 1,250 DEGs (1,094 upregulated and 156 downregulated) were obtained from the aging dataset (Figure 2C). An intersection was conducted and we obtained 563 common DEGs (190 upregulated and 373 downregulated) between the pain datasets (Figures 2D,E). After excluding genes with opposite expression trends, we performed a Venn diagram analysis. Finally, 16 co-DEGs (13 upregulated and 3 downregulated) were identified (Figures 2F,G and Table 1).

**TABLE 1:** 16 co-DEGs between neuropathic pain and aging.

Gene	Terms	Pain1		Pain2		Aging	
				
		LogFc	adj.P.Val	LogFc	adj.P.Val	LogFc	P.Val
Cxcl14	C-X-C motif chemokine ligand 14	3.66	8.73E-06	3.43	1.26E-04	2.19	2.36E-04
Gpnmb	Glycoprotein nmb	2.03	4.75E-05	0.62	1.21E-02	1.57	5.05E-04
Bmp7	Bone morphogenetic protein 7	0.72	6.79E-03	1.57	2.49E-05	1.19	8.29E-04
Ramp1	Receptor activity modifying protein 1	0.87	2.49E-05	1.23	1.94E-04	0.87	6.51E-03
Lilrb4	Leukocyte immunoglobulin like receptor B4	1.53	4.72E-03	1.31	5.77E-06	0.63	9.95E-03
Reg3b	Regenerating islet-derived 3 beta	1.81	4.69E-06	2.52	1.59E-02	1.67	9.99E-03
Cfd	Complement factor D	0.84	1.60E-03	1.74	8.95E-03	1.47	1.60E-02
Serpinb2	Serpin family B member 2	2.98	7.40E-04	1.84	1.68E-09	2.44	1.97E-02
Clec4a3	C-type lectin domain family 4, member a3	1.45	1.02E-05	0.99	2.91E-02	0.60	2.30E-02
Serping1	Serpin family G member 1	1.04	2.36E-03	0.95	1.59E-02	1.07	2.44E-02
RT1-Da	RT1 class II, locus Da	0.86	4.37E-02	2.01	9.06E-05	0.86	2.99E-02
Fcgr2b	Fc gamma receptor IIb	3.02	2.48E-03	1.82	4.93E-05	0.96	3.31E-02
Fblim1	Filamin binding LIM protein 1	0.63	4.63E-02	0.65	4.63E-03	1.50	3.58E-02
Abcd2	ATP binding cassette subfamily D member 2	–1.08	8.68E-05	–0.97	1.85E-06	–0.75	1.39E-02
Hs3st2	Heparan sulfate-glucosamine 3-sulfotransferase 2	–1.47	8.92E-06	–1.97	5.34E-05	–0.73	3.70E-02
Rem2	RRAD and GEM like GTPase 2	–1.69	8.04E-05	–1.06	3.59E-02	–0.89	9.97E-03

### Tissue-most expressed gene analysis

To better identify tissue- and organ-specific expressed co-DEGs, we searched our co-DEGs in BioGPS. The most highly tissue-related expression system was the hematologic/immune system. The neurologic systems, including pineal, ventral striatum, and primary cortical neurons ranked second. Furthermore, the digestive system and others like the cornea ranked third, while the circulatory system and urinary had another similar level of enrichment (Table 2). The results indicated the possible related systems across pain and aging.

**TABLE 2:** Tissue-most expressed gene identified by BioGPS.

System	Tissue/Cell	Gene
Hematologic/immune	Spleen, thymus, bone marrow	Fcgr2b, RT1-Da, Reg3b, Cfd
Circulatory	Endothelial cell	Fblim1
Neurologic	Pineal, ventral striatum, primary cortical neurons	Serpring1, Cxcl14, Bmp7
Digestive	Small intestine, large intestine	Reg3b, Fblim1
Urinary	Kidney	Serping1
Others	Cornea	Cxcl14, Bmp7

### Functional enrichment pathway analysis

**FIGURE 3:**
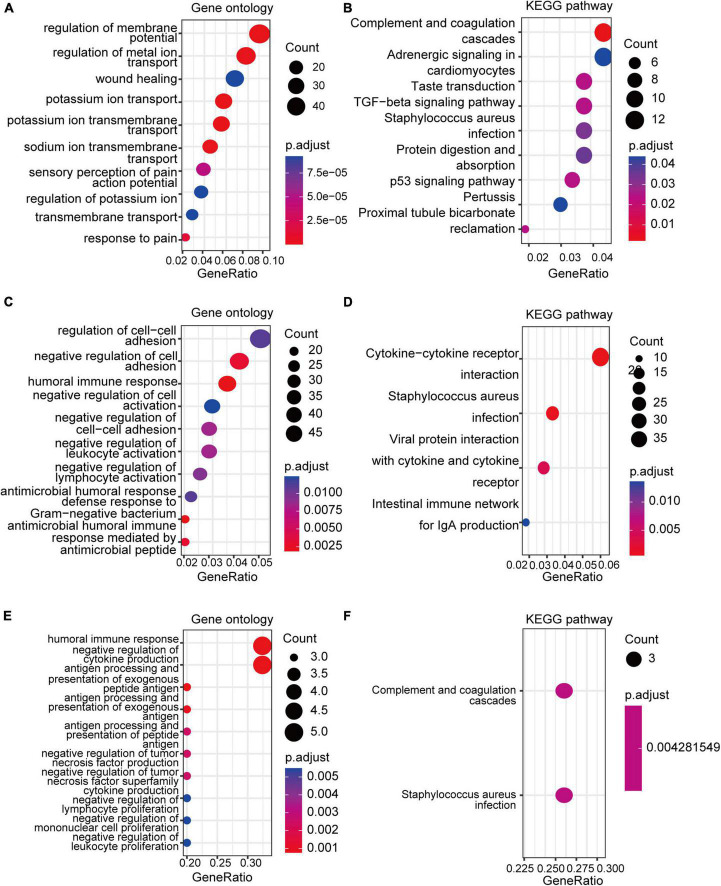
GO and KEGG analyses of the DEGs and co-DEGs. **(A,B)** GO analysis and KEGG pathways analysis of the co-DEGs of the pain datasets. **(C,D)** GO analysis and KEGG pathways analysis of the DEGs of the aging dataset. **(E,F)** GO analysis and KEGG pathways analysis of the co-DEGs between the pain and aging datasets. GO, Gene Ontology; KEGG, Kyoto Encyclopedia of Genes and Genomes; DEGs, differential expressed genes; co-DEGs, common differential expressed genes.

GO and KEGG enrichment analyses of the DEGs and co-DEGs above were performed in R using the “clusterProfiler” package. Results showed that DEGs in pain datasets were mainly enriched in membrane potential, metal ion transport, wound healing, sensory perception of pain, action potential, transmembrane transport, complement, and coagulation cascades etc. (Figures 3A,B and Supplementary Tables 1, 2), while the DEGs in the aging dataset were mainly enriched in regulation of cell-cell adhesion, negative regulation of cell activation, cytokine-cytokine receptor interaction etc. (Figures 3C,D and Supplementary Tables 3, 4). Further functional enrichments analyses indicated that the co-DEGs between pain and aging were principally associated with humoral immune response, negative regulation of cytokine production, antigen processing, and presentation of exogenous peptide antigen, antigen processing, and presentation of exogenous antigen, antigen processing and presentation of peptide antigen, negative regulation of tumor necrosis factor production, negative regulation of tumor necrosis factor superfamily cytokine production, negative regulation of lymphocyte proliferation, negative regulation of mononuclear cell proliferation, and negative regulation of leukocyte proliferation (Figure 3E and Supplementary Table 5). Furthermore, two KEGG pathways were identified, including the complement cascade and Staphylococcus aureus infection (Figure 3F and Supplementary Table 6).

**FIGURE 4:**
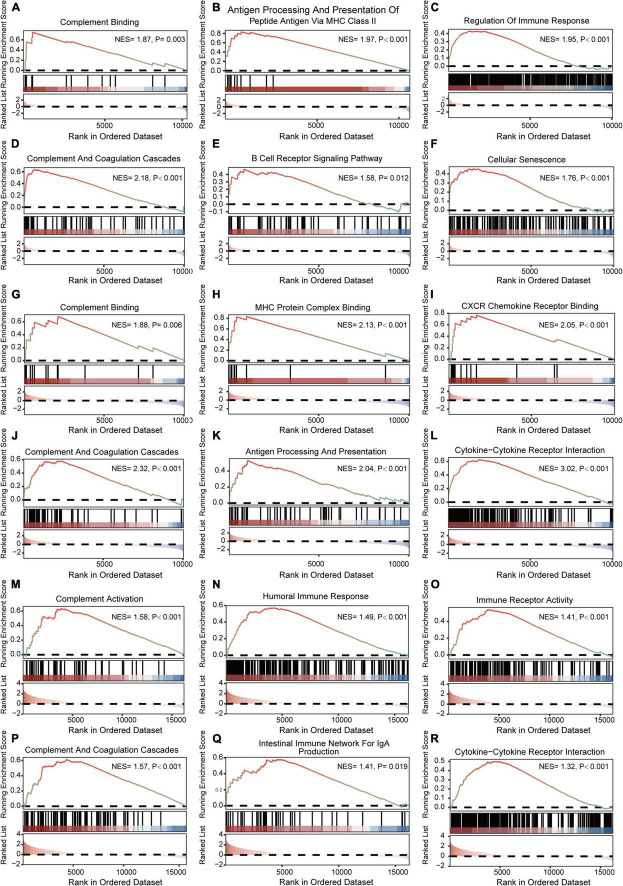
GSEA enrichment analyses of GSE24982, GSE63442, and GSE63651. **(A–C)** Immunity-related GO items enriched in pain based on GSE24982. **(D–F)** Immunity-related KEGG pathways and cellular senescence enriched in pain based on GSE24982. **(G–I)** Immunity-related GO items enriched in pain based on GSE63442. **(J–L)** Immunity-related KEGG pathways enriched in pain based on GSE63442. **(M–O)** Immunity-related GO items enriched in aging based on GSE63651. **(P–R)** Immunity-related KEGG pathways enriched in aging based on GSE63651. GSEA, Gene set enrichment analysis; NES, Normalized enrichment score; P, *p*-value.

The results of GSEA analyses consistently indicated the importance of immune regulation across pain and aging. Complement binding (NES = 1.87, *P* = 0.003; NES, normalized enrichment scores; P, *P*-value), antigen processing and presentation of peptide antigen *via* MHC class II (NES = 1.97, *P* < 0.001), regulation of immune response (NES = 1.95, *P* < 0.001), complement and coagulation cascades (NES = 2.18, *P* < 0.001) and B cell receptor signaling (NES = 1.58, *P* = 0.012) pathway were significantly enriched in pain based on GSE24982 (Figures 4A–E). The cellular senescence item was also upregulated considerably (NES = 1.76, *P* < 0.001) (Figure 4F). Meanwhile, Complement binding (NES = 1.88, *P* = 0.006), MHC protein complex binding (NES = 2.13, *P* < 0.001), CXCR chemokine receptor binding (NES = 2.05, *P* < 0.001), complement and coagulation cascades (NES = 2.32, *P* < 0.001), antigen processing and presentation (NES = 2.04, *P* < 0.001) and cytokine—cytokine receptor interaction (NES = 3.02, *P* < 0.001) were enriched in pain based on GSE63442 (Figures 4G–L). Complement activation (NES = 1.58, *P* < 0.001), humoral immune response (NES = 1.49, *P* < 0.001), immune receptor activity (NES = 1.41, *P* < 0.001), complement and coagulation cascades (NES = 1.57, *P* < 0.001), intestinal immune network for IgA production (NES = 1.41, *P* = 0.019), and cytokine—cytokine receptor interaction (NES = 1.32, *P* < 0.001) were enriched in aging based on GSE6365 (Figures 4M–R).

### Protein-protein interaction network construction and analysis

**FIGURE 5:**
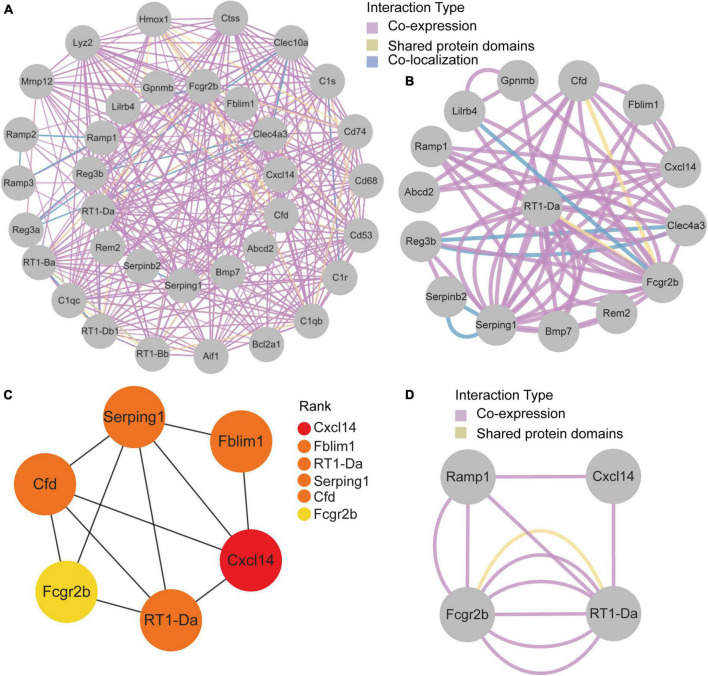
Protein-Protein Interaction network of co-DEGs. **(A)** co-DEGs and their co-expression genes were analyzed using GeneMANIA database. The 20 most frequently changed neighboring genes are shown in the outer circle. co-DEGs are shown in the inner circle. Each node represents a gene. The line color represents the types of network interaction. **(B)** co-DEGs interaction network. The line color represents the types of network interaction. **(C)** Top 6 hub genes identified by cytoHubba plug-in. Nodes with different color represent different rank. **(D)** A significant cluster module extracted by MCODE. Each node represents a gene. The line color represents the types of network interaction.

GeneMANIA database was first used to perform the PPI network construction. From the results, 15 co-DEGs except Hs3st2, and 20 related genes with 950 total links were demonstrated. Approximately 95.10% of the interactions were based on co-expression data, 3.11% of them were based on shared protein domains, while only 1.8% of them were predicted based on co-localization. The networks of co-DEGs and their co-expressed genes were drawn (Figure 5A). We further extracted co-DEGs networks with interactions (Figure 5B). From the results, 15 co-DEGs except Hs3st2 showed a significant interaction, most of which were based on co-expression data. Plug-in cytoHubba was then used to identify the potential hub genes. We further screened 6 hub genes including Cxcl14, Fblim1, RT1-Da, Serping1, Cfd, and Fcgr2b (Figure 5C). MCODE was then applied to identify potential key gene cluster modules. The result demonstrated one connected gene module (Figure 5D) containing 4 co-DEGs, including Ramp1, Cxcl14, Fcgr2b, and RT1-Da.

### Immune infiltration analysis

**FIGURE 6:**
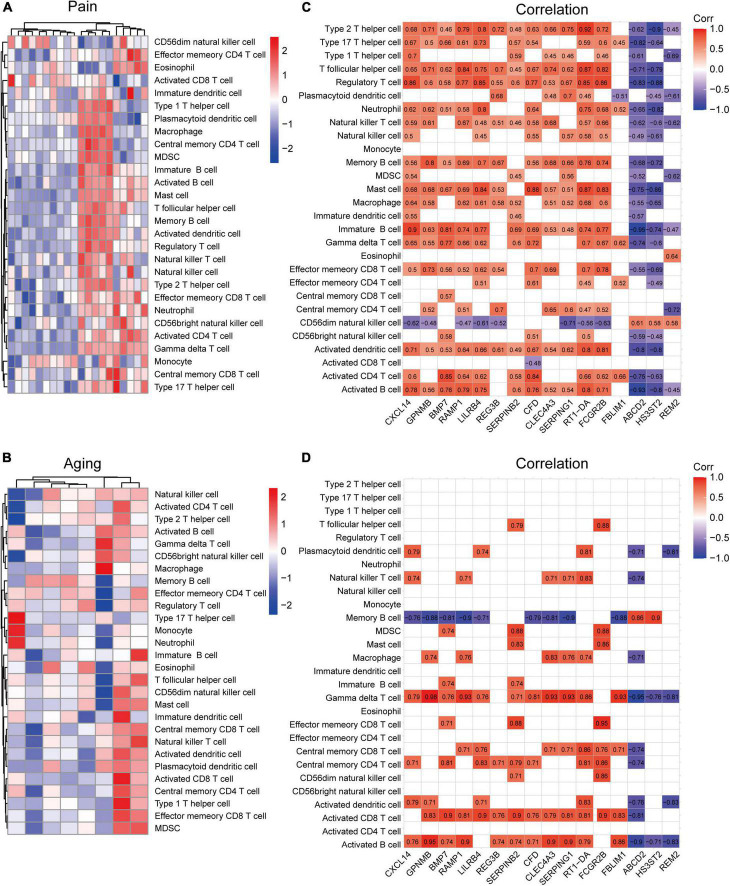
Immune infiltration and correlations with co-DEGs. **(A)** Relative infiltration abundance of 28 immune cell subpopulations in pain. **(B)** Relative infiltration abundance of 28 immune cell subpopulations in aging. **(C)** The correlations between co-DEGs and immune cells infiltration in pain. **(D)** The correlations between co-DEGs and immune cells infiltration in aging.

Based on the GSE24982 and GSE63651 datasets, we applied the ssGSEA method to decode the relative infiltration abundance of 28 immune cell subpopulations in pain and normal controls as well as aging and normal controls. Compared with normal controls, both pain and aging group exhibited a higher infiltration abundance of most immune cells, suggesting a microenvironment of immune activation (Figures 6A,B). Among the results, activated B cell, mast cell, activated dendritic cell, CD56 bright natural killer cell, effector memory CD8 + T cell and type 2 T helper cell were consistently up-regulated in the pain and aging condition. Moreover, correlations between co-DEGs and 28 immune cells were shown. We were surprised to find that hub genes here correlate simultaneously with the activated B cell, activated dendritic cell, Gamma delta T cell, central memory CD4 + T cell and mast cell in pain and aging diseases (Figures 6C,D), suggesting their immunity roles across the disorders.

### Target transcription factors and microRNAs prediction and network construction

**FIGURE 7:**
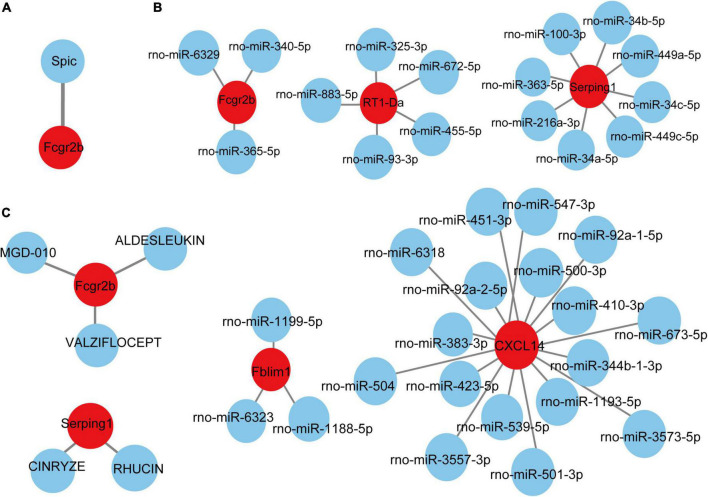
TF-miRNA-drugs regulatory networks. **(A)** TF prediction for hub genes based on TRRUST database. **(B)** miRNA network prediction for hub genes based on miRDB database. **(C)** Drug-genes interaction network prediction. Red nodes represent hub genes. Blue nodes represent predicted TFs, miRNA or drugs.

Based on the TRRUST database, Spic, a TF (Figure 7A) that may regulate the gene Fcgr2b was identified. According to the miRDB database, 36 target miRNAs were also predicted to interact with genes (Figure 7B). The TF-genes and miRNA-genes interaction networks were visualized by Cytoscape software.

### Prediction of targeted drugs

According to the DGIdb database, 5 potential drugs (Aldesleukin, Valziflocept, MGD-010, Cinryze, and Rhucin) were predicted to target the hub genes (Figure 7C). Cytoscape was used to visualize the drug-genes interaction.

## Discussion

Nowadays, many studies have confirmed the correlation between neuropathic pain and aging. The incidence of pain gradually increases with aging (Koc and Kutsal, 2015). Meanwhile, many studies have shown that the occurrence of pain may accelerate the progression of aging. Miaskowski et al. (2020) showed that older adults with chronic pain in multiple locations have a significantly increased risk of developing disability over time and are at risk for decreased mobility. Morrison and his colleagues revealed that many inflammatory factors, such as tumor necrosis factor-α (TNF-α), nuclear transcription factors (NF-κB), and interleukin-6 (IL-6), are both significantly correlated with chronic pain and aging, which play an important role in pain sensitization and cell senescence (Morrison et al., 2013). However, the direct link and specific mechanisms remain unclear. Therefore, it’s urgent to explore the molecular mechanisms and pinpoint the particular links between pain and aging.

Our study was conducted based on three datasets, two of which were associated with pain while another was related to aging. We first analyzed the DEGs in each pain dataset and took the intersection after eliminating the opposite trend of the DEGs in the two datasets, and then we obtained the common DEGs of pain. Then we analyzed the aging dataset and followed the same method as above to take the intersection. Finally, we identified 16 co-DEGs in pain and aging datasets (13 upregulated and 3 downregulated).

BioGPS was an online tool that created as a centralized gene-annotation portal for clustering distributed genes (Wu et al., 2009). It has been widely used to explore tissue- and organ-specific genes, indicating the correlated systems in diseases (Fang et al., 2022; Li et al., 2022; Xing et al., 2022). According to BioGPS, the co-DEGs above were mainly expressed in the hematologic/immune system and neurologic system, which highlights the immune system’s role in neuropathic pain and aging. However, though many studies have also indicated the potential significance of immune response in neuropathic pain and aging (Scholz and Woolf, 2007; Kavelaars and Heijnen, 2021), the underlying mechanisms remain to be further elucidated.

We performed a series of bioinformatic analyses to investigate the function of the DEGs. According to GO and KEGG enrichment analyses, DEGs in pain datasets were majorly enriched in regulating membrane potential, metal ion transport, wound healing, sensory perception of pain, action potential and transmembrane transport, consistent with the fact that pain is mainly associated with sensory conduction and ion transport. DEGs in aging were enriched in regulation of cell-cell adhesion, humoral immune response, negative regulation of cell activation, and antimicrobial humoral response, which showed that aging is linked to immune response and the activation of immune cells. Most importantly, we found that the co-DEGs in both pain and aging datasets were mainly enriched in immune function and pathways. KEGG pathway analysis demonstrated that the co-DEGs were enriched in complement and coagulation cascades and Staphylococcus aureus infection, which is primarily related to immunity and inflammation.

Further GSEA enrichment analysis also validated the importance of immune regulation in pain and aging. The results showed that complement binding, antigen processing and presentation, complement and coagulation cascades, B cell receptors, MHC protein complex binding, CXCR chemokine, and cytokine receptors were mainly enriched in pain conditions. Besides, it was worth noting that cellular senescence, a hallmark of aging (Childs et al., 2015), was also activated in pain, indicating the correlation between pain and aging. Meanwhile, complement activation, humoral immune response, immune receptor activity and complement and coagulation cascades were upregulated in aging. As the hypersensitivity caused by inflammatory immune response is one of the crucial factors of neuropathic pain (Li et al., 2020), and immune dysregulation has also emerged as a vital component of aging (Borgoni et al., 2021), we speculate that immune homeostasis may play a crucial role across pain and aging. Therefore, immune targeting therapies might be a potent approach for solving pain and aging. It was different from previous studies that we here paid extra attention to the functional analyses of the co-DEGs across the pain and aging datasets, thus providing new ideas for mechanisms and treatments for the comorbidity of the two diseases.

Subsequently, we constructed the PPI network and applied CytoHubba and MCODE plug-ins to identify the hub genes and critical modules. Cxcl14, Fblim1, RT1-Da, Serping1, Cfd, and Fcgr2b were further screened. It’s worth noting that these genes were upregulated in both pain and aging datasets, suggesting their essential roles in pain and aging.

Cxcl14 (C-X-C motif chemokine ligand 14) is a CXC chemokine ligand that constitutively expressed in immune cells and throughout the central nervous system. Lu et al. (2016) have summarized its significant role in immunity as an emerging immune and inflammatory modulator. It has been reported that deletion of Cxcl14 in mice could accelerate skeletal myogenesis by promoting cell cycle withdrawal, which may be a promising target for developing therapeutics to treat aging-related muscle wasting (Waldemer-Streyer et al., 2017). And it has also been identified as a candidate marker for endometrial aging (Kawamura et al., 2021). Besides, cxcl14 is an essential factor in the initial and maintenance of pain. Previous studies showed that cxcl14 contributes to the modulation of somatosensation in concert with somatostatin (Yamamoto et al., 2020). And it has been demonstrated that NFATc2-dependent epigenetic upregulation of cxcl14 is involved in the development of neuropathic pain induced by paclitaxel (Liu et al., 2020). Moreover, a microarray analysis of rat sensory ganglia after local inflammation also implicates the significant upregulation of cxcl14 in pain (Strong et al., 2012).

Fblim1 (Filamin binding LIM protein 1) encodes a protein with an N-terminal filamin-binding domain, a central proline-rich domain, and multiple C-terminal LIM domains that can serve as an anchoring site for cell-ECM adhesion proteins and filamin-containing actin filaments. It has been implicated in the pathogenesis of sterile bone inflammation (Cox et al., 2017). Meanwhile, migfilin, a protein encoded by Fblim1, is widely expressed in different adherent and circulating blood cells and can regulate integrin activation in naturally occurring vascular cells, endothelial cells and neutrophils. Besides, tightly regulated expression of migfilin is also essential for neuronal development (Ishizuka et al., 2018). However, the role of Fblim1 in the pathogenesis of pain and aging remains unknown.

RT1-Da (RT1 class II, locus Da) is a protein-coding gene that enables T cell receptor binding activity, MHC class II protein complex binding activity and peptide antigen-binding activity. Prior studies have shown that RT1-Da is significantly associated with diseases mediated by the immune system (Vestberg et al., 1998). And it was also identified as an intermediate marker for bilateral hippocampal response to mild traumatic brain injury (Almeida-Suhett et al., 2014).

Serping1 (Serpin Family G Member 1) encodes a highly glycosylated plasma protein regulating the complement cascade. Its protein product inhibits activated C1r and C1s of the first complement component, thus regulating complement activation (Murray-Rust et al., 2009). It has been demonstrated to be associated with hereditary angioneurotic edema (Canonica and Rossi, 2012).

Cfd (Complement Factor D) encodes a member of the S1, or chymotrypsin, family of serine peptidases. As a serine protease of the alternative complement pathway, Cfd is required to form C3 convertase, which is the rate-limiting enzyme (Barratt and Weitz, 2021). Mutations in Cfd might result in the deficiency of complement factor D, which is associated with recurrent bacterial meningitis infections in human patients (Wiles et al., 2020).

Fcgr2b (Fc Gamma Receptor IIb) is a protein-coding gene associated with Systemic Lupus Erythematosus and Malaria (Su et al., 2004). It has been reported to play an important role in the B cell receptor signaling pathway and immune response. Evidence has suggested that Fcgr2b is closely associated with arthritis-related joint pain (Bersellini Farinotti et al., 2019), anterior cingulate cortex and prefrontal cortex alteration after nerve injury (Poh et al., 2012; Zhang et al., 2022), and stroke in aged rats (Buga et al., 2012). However, few studies have demonstrated its role in the interconnection between pain and aging. Our work further highlights its significant role in pain and aging interconnection.

Neuroinflammation is one of the crucial factors promoting the occurrence of neuropathic pain. It is usually characterized by the activation and infiltration of leukocytes, the activation of glial cells, and the increasing inflammatory mediators (Ji et al., 2016). Meanwhile, immunity and inflammation were also dispensable in aging (Ovadya et al., 2018; Wu et al., 2019). For example, senescent cells usually accelerate aging phenotypes through a senescence-associated secretory phenotype (SASP), inducing amounts of inflammatory mediators (IL-1β, IL-6, and TNF-α, etc.). It has also been proved that some critical cytokines and immune cells in aging are indeed neuromodulators of the peripheral and central nervous systems (Atzeni et al., 2019; Durante et al., 2021). Consistently, we found that in both pain and aging condition, there exhibited a higher infiltration abundance of most immune cells, including the activated B cell, mast cell, activated dendritic cell, CD56 bright natural killer cell, effector memory CD8 + T cell and type 2 T helper cell, suggesting a microenvironment of immune activation. Furthermore, we were surprised that those hub genes simultaneously demonstrated a close correlation with the activated B cell, activated dendritic cell, Gamma delta T cell, central memory CD4 + T cell and mast cell in pain and aging diseases, indicating their importance across pain and aging.

TFs are proteins that can bind to DNA and regulate the transcription of DNA into RNA by attaching to the transcription factor binding site, representing the point of convergence of multiple signaling pathways within eukaryotic cells (Kontos et al., 2013). MiRNAs are a class of small and non-coding RNAs that function as post-transcriptional gene regulators by inducing mRNA degradation or repressing mRNA translation (He and Hannon, 2004). The roles of TFs and microRNAs in the pathophysiology of pain and aging have been emerging (Andersen et al., 2014; Zhang and Chi, 2018; Kinser and Pincus, 2020). Here, we constructed TFs- and miRNAs- related regulatory networks for hub genes and module-related key genes to show the potential interactions under pain and aging conditions. As shown on the network, we obtained Spic (Spi-C Transcription Factor) as the key regulatory factor for gene Fcgr2b expression. Besides, 36 miRNAs were predicted to regulate the above genes.

Based on the DGIdb database, drug compounds were put forward. Aldesleukin, Valziflocept, MGD-010, Cinryze, and Rhucin were highlighted among the potential effective drugs. Aldesleukin is an interleukin-2 that FDA approved for the treatment of adults with metastatic renal cell carcinoma (metastatic RCC) and metastatic melanoma (Clement and McDermott, 2009). Song et al. (2002) found that IL-2 can produce analgesic effects in morphine-insensitive mice (Song et al., 2002). Zhang et al. (2020b) found that low doses of IL-2 can also effectively prevent and reverse behavioral development associated with acute and chronic headaches. And IL-2 has also been reported in modulating the inflammation-related senescence of hippocampal neurons. Considering the significance of IL-2, we speculated that Aldesleukin might have a potential value in treating pain and aging. Valziflocept is a recombinant soluble FcγIIb receptor that targets Fc and FcγR (Zuercher et al., 2019). FcγR has been reported as a potential therapeutic target for pain in autoimmune diseases (Bersellini Farinotti et al., 2019; Peyrin-Biroulet et al., 2019), and it was also crucial in aging neutrophils (Gasparoto et al., 2021). There is no doubt that Valziflocept may benefit patients with comorbidities of pain and aging to some extent. MGD-010 is a DART (Dual Affinity Retargeting) antibody molecule with great potential in autoimmune disease treatment, which can downregulate B cell receptors and finally reduces signaling. As ssGSEA indicated the upregulation of activated B cells in pain and aging, MGD-010 might also be an effective drug in pain and aging by targeting B cell. Besides, Rhucin, and Cinryze are C1 esterase inhibitors that target hereditary angioedema with FDA approval (Gompels and Lock, 2011). And Chen et al. (2013) found that neutralizing antibodies against the globular heads of the complement protein’s C1q receptor (gC1qR) could also alleviate mechanical hyperalgesia in rats with partial sciatic nerve ligation. Though the efficacy of predictive drugs needs further validation, it provides a new direction that targeting the hub genes *via* various mechanisms may help alleviate pain and aging conditions.

Nevertheless, there are still several issues to be addressed starting from our preliminary evidence. Firstly, it is a retrospective study that requires more extensive data analysis before addressing external validation. Secondly, some predictive results are limited in significance due to the knowledge base currently available to database users. Thirdly, the expressions of the hub genes and their roles in pain and aging need to be further explored and assessed. Finally, detailed molecular mechanisms and the regulatory networks still lack research efforts to establish characterization *in vitro* and *in vivo*.

## Conclusion

This is the first study using integrated transcriptome analyses to explore the relationship between neuropathic pain and aging. We put forward ideas to explore and identify the co-DEGs, hub genes to support our understanding of their potential roles in the immune microenvironment, and find specific players among TFs, miRNAs and potential drugs in neuropathic pain and aging. Humoral immune response, especially the regulation of the immune cells like activated B cells, might be the critical mechanism of the interaction. Cxcl14, Fblim1, RT1-Da, Serping1, Cfd, and Fcgr2b might play a significant role in the process. Spic, miR-883-5p, miR-363-5p et al. and potential drugs including Aldesleukin, Valziflocept, MGD-010, Cinryze, and Rhucin were further predicted. In conclusion, our work proposed to explore the correlation between pain and aging, and the preliminary findings highlighted a feasible strategy to identify potential co-DEGs targets addressing early diagnosis and therapy aspects.

## Data availability statement

The datasets presented in this study can be found in online repositories. The names of the repository/repositories and accession number(s) can be found in the article/Supplementary material.

## Author contributions

WZ and JX designed the study. QY, ZH, and WL collated the data, carried out data analyses, and produced the initial draft of the manuscript. FY and WZ helped perform the analysis with constructive discussions. All authors have read and approved the final submitted manuscript.

## Footnotes

1
http://www.ncbi.nlm.nih.gov/geo

2
http://biogps.org

3
http://genemania.org/search/

4
https://www.grnpedia.org/trrust/

5
http://mirdb.org/

6
https://dgidb.org/
